# Amniocentesis is a low-risk procedure in HIV-treated pregnant women

**DOI:** 10.1186/1758-2652-13-S4-P163

**Published:** 2010-11-08

**Authors:** MM Simoes, CO Marques, F Albergaria, C Guerreiro, A Pereira, J Correia, J Castela

**Affiliations:** 1 Maternidade Dr. Alfredo da Costa, Via paralela a Avenida Lusiada, Lisboa, Portugal

## Background

The iatrogenic risk of HIV vertical transmission, calculated in initial epidemiologic studies of this infection seemed to contra-indicate the performance of invasive prenatal diagnosis (PND) techniques. The implementation of highly active antiretroviral therapy (HAART) represented a turning point in PND management, owing to a rapid and effective reduction of the maternal viral load.

## Purpose of the study

To evaluate the risk of vertical transmission in pregnant women infected with HIV, submitted to amniocentesis during the second trimester of pregnancy.

## Methods

Analysis of the amniocentesis performed in our institution, in pregnant HIV positive (N=23). The sample was obtained from the database which included all HIV-infected pregnant woman who gave birth between 1996 and 2009 (n= 731), in our institution. Data were collected in order to obtain: demographic characteristics of the sample, HIV subtype and its transmission category, antiretroviral therapy, gestational age, indication of amniocentesis, viral load and T CD4+ lymphocyte count determined close to performing amniocentesis and close to labour, result of the chromosomal analysis, obstetrical complications, type of labour and data referring to the newborn. Our sample was divided in two subgroups: one comprising women with adequate pregnancy surveillance in our immunodepression unit (Group A, n=17) and other comprising women with the diagnosis of HIV infection after performing amniocentesis, with no surveillance or therapy until the amniocentesis (Group B, n=6).

## Summary of results

Among the 23 newborns, only one case of HIV 1 infection was diagnosed, in group B. It occurred in a patient with a diagnosis of HIV infection at 30 weeks gestation, after being submitted to amniocentesis at 16 weeks gestation for primary CMV maternal infection, and she had a vaginal delivery at 38 weeks. Antiretroviral therapy was not accomplished as well as adequate pregnancy surveillance.

## Conclusions

When there is an indication to perform an amniocentesis in a pregnant woman infected with HIV, it is legitimate to perform it if the woman is following a HAART, and is ideally under viral suppression. Although the number of our sample is limited, there was no case of vertical transmission among pregnant women with adequate pregnancy surveillance, who were submitted to amniocentesis under HAART. It would be extremely important to analyse wider results, in a multicentric study.

